# Association of socioeconomic status in childhood with major depression and generalized anxiety disorder: results from the World Mental Health Japan survey 2002–2006

**DOI:** 10.1186/1471-2458-14-359

**Published:** 2014-04-14

**Authors:** Manami Ochi, Takeo Fujiwara, Rie Mizuki, Norito Kawakami

**Affiliations:** 1 Department of Social Medicine, National Research Institute for Child Health and Development, 2-10-1 Okura, Setagaya-ku, Tokyo 157-8535, Japan; 2 Department of Developmental Social Medicine, Mie University Graduate School of Medicine, Mie, Japan; 3 Department of Mental Health, Graduate School of Medicine, The University of Tokyo, Tokyo, Japan

**Keywords:** Childhood environment, Socioeconomic status, Mental health, Depression, Anxiety, Gender

## Abstract

**Background:**

Low socioeconomic status (SES) in childhood is known to be a significant risk factor for mental disorders in Western societies. The purpose of this study was to investigate whether a similar association exists in Japan.

**Methods:**

We used data from the World Mental Health Japan Survey conducted from 2002–2006 (weighted N = 1,682). Respondents completed diagnostic interviews that assessed lifetime prevalence of major depression (MD) and generalized anxiety disorder (GAD), as defined by the Diagnostic and Statistical Manual of Mental Disorders, Fourth Edition. Associations between parental education (a proxy of SES in childhood) and lifetime onset of both disorders were estimated and stratified by gender using discrete-time survival analysis.

**Results:**

Among women, high parental education was positively associated with MD (odds ratio [OR]: 1.81, 95% confidence interval [CI]: 1.03-3.18) in comparison with low parental education, even after adjustment for age, childhood characteristics, and SES in adulthood. This same effect was not found for men. In contrast, higher parental education was associated with GAD (OR: 6.84, 95% CI: 1.62-28.94) in comparison with low parental education among men, but this association was not found among the women, in the fully adjusted model.

**Conclusions:**

In Japan, childhood SES is likely to be positively associated with the lifetime onset of mental disorders, regardless of family history of mental disorders, childhood physical illness, or SES in adulthood. Further study is required to replicate the current findings and elucidate the mechanism of the positive association between mental disorders and childhood SES.

## Background

It is widely known that low socioeconomic status (SES) is associated with psychological problems such as depression and anxiety disorders [1-5]. This association can be explained in two ways: (1) low SES actually induces a mental disorder (social causation); or (2) mental disorders limit employment opportunities, causing individuals to fall into the low SES category (health selection) [6,7].

Previous studies have shown that SES in childhood has a direct effect on the development of mental disorders later in life [8-15]. For example, Gilman et al. reported that participants whose parent was engaged in manual labor either at the time of their birth or when they were seven years old were significantly more likely to develop major depression (MD) in their lifetime, even after adjusting for SES in adulthood [11]. However, since most of these studies were performed in Western countries, it is uncertain whether a similar association exists in Japan, where SES likely affects mental disorders differently [16,17]. For instance, while education has been found to be inversely associated with depression in the USA, no such association has been found in Japan [16].

MD and generalized anxiety disorder (GAD) must be addressed in particular, in view of their high prevalence [18,19]. The lifetime prevalence of MD and GAD in the US is 16.6% and 5.7%, respectively in 2001–2003 [18], and in Japan, 4.4% for MD in 2005 [20]. Because MD and GAD are associated with several major causes of death, such as suicide [21] or cardiovascular disease [22,23], and greater disability-adjusted life years [24], further prevention efforts are needed. An investigation into the associations between childhood SES and MD or GAD may provide crucial information concerning the possible etiologies of these disorders. Further, by stratifying the data according to gender, the higher prevalence of these disorders among women may be explained [11].

Against these backgrounds, we hypothesized that childhood SES is associated with the lifetime onset of mental disorders, regardless of family history of mental disorders, childhood physical illness, or SES in adulthood, based on life-course epidemiology [25]. By focusing on SES in childhood, we can include the early onset cases, which are usually excluded in studies of the association between SES in adulthood and mental disorders in order to avoid reverse causation [26]. Thus, the purpose of this study was to investigate whether SES in childhood was associated with MD and GAD in both adult men and women.

## Methods

### Sample

Data from the World Mental Health Japan (WMHJ) Survey conducted between 2002 and 2006 were used. The WMHJ conducted an epidemiological survey of Japanese people aged 20 years and older as part of the World Health Organization’s World Mental Health Survey Initiative [27]. Details of the WMHJ survey design, sampling, and field procedures have been described in previous research [28].

Three urban cities and eight rural municipalities in Japan were selected as study sites. These sites were selected because of their geographic variation, the availability of site investigators, and the cooperation of local government officials. Participants were randomly selected from a pool of eligible voters (i.e., registered residents) aged 20 years or older.

An internal sampling strategy was used to reduce respondent burden by dividing the interview into two parts. Part I included a core diagnostic assessment (details given below) and obtained the demographic variables of all the respondents. Part II included questions about risk factors, including childhood SES. Part II was administered to 1,682 of the 4,134 individuals who responded to the questionnaire in Part I (including all respondents with one or more lifetime disorders, as well as a probability subsample of approximately 25% of the other respondents). The total response rate was 55.1%. This sampling method was not significantly different from those used in the World Mental Health Surveys conducted in other countries [29].

The data were weighted to adjust for differential probabilities of selection and non-response (Weighted N = 1,682; N [men] = 734; N [women] = 948). Details of sample weights have been reported previously [19]. Sample size was calculated by assuming the lifetime prevalence of mental disorders to be between 5 and 10% [29] in low and high childhood SES groups with equal distribution ratios (with a Type I error = 0.05 and Type II error = 0.2), respectively. This yielded a figure of 948 participants who were able to successfully complete this study.

Written consent was obtained from every respondent at all study sites. The survey recruitment, consent, and field procedures were approved by The Human Subjects Committees of Okayama University Graduate School of Medicine, Dentistry, and Pharmaceutical Sciences, the Japan National Center of Neurology and Psychiatry, Nagasaki University’s Graduate School of Biomedical Sciences, Yamagata University’s Graduate School of Medical Science, and Juntendo University’s Graduate School of Medicine.

### Diagnostic assessment

The WMHJ used a Japanese-translated, computer-assisted version of the World Health Organization Composite International Diagnostic Interview, Version 3.0 (WHO-CIDI 3.0) to assess mental disorders in individuals according to the Diagnostic and Statistical Manual of Mental Disorders, Fourth Edition [27]. Details concerning the translation process from English to Japanese have been reported previously [19]. Lifetime diagnoses of MD and GAD were approximated by the presence or absence of diagnoses of these disorders that respondents admitted to having, up to the time of the interviews. Diagnostic hierarchy and organic exclusion rules were used for making diagnoses.

The CIDI retrospectively assessed the age of onset for the disorders; however, in view of the existing evidence that retrospective age-of-onset reports are often biased [30], a special question sequence (previously used in the National Comorbidity Survey Replication) was introduced to improve the accuracy of reporting. In brief, the age of onset reported by the respondents was confirmed by other sequential questions, such as “Was it before you went to school?”. Onset age was set at the upper end of the bound of uncertainty (e.g., age: 12 years for respondents who reported that onset was before their teenage years). Previous research has shown that this question sequence yields more credible responses than do standard age-of-onset questions [31].

### Socioeconomic status in childhood

SES in childhood was measured using the proxy variable of parents’ education, because parental education is usually determined before the birth of the respondent; thus, we can use this measure to assess the impact of childhood SES on the lifetime incidence of MD or GAD. The number of years of education for both parents was surveyed, and the responses were categorized into three groups: less than a high school (0–11 years), high school (12 years), and some college or more (≥13 years). If the number of years of education was unknown, this became a dummy variable. If a respondent’s parents’ years of education were in discord, we used the higher number of years as parental education for our study.

### Covariates

Under the assumption that they could be possible confounders or mediators in the relationship between childhood SES and lifetime onset of MD and GAD, we assessed data on certain childhood characteristics and SES in adulthood. The childhood characteristics of interest included parental mental illness and the presence of personal physical illness in the respondent’s childhood (based on responses to yes/no questions). SES in the respondents’ adulthood was measured by the individual’s number of years of education, categorized into less than high school (0–11 years), high school (12 years), some college (13–15 years), and college or more (≥16 years). Further, the respondent’s current annual household income was categorized with reference to the poverty line in Japan [32,33], as either low (<3 million yen), middle (3–9.9 million yen), or high (≥10 million yen).

### Analysis methods

The models were estimated in a discrete-time survival framework with person-years as the unit of analysis. The obtained person-oriented data set (containing information on the age of onset for each mental disorder) from the cross-sectional survey was converted into a person-period dataset (containing information on each discrete time period for the individual, censoring the onset of each mental disorder) [34]. Each model was controlled for person-years, age category, and covariates. The survival coefficients and their standard errors (SEs) in the best-fitting model were exponentiated and are reported in the form of odds ratios (OR) and 95% confidence intervals (CI).

Model 1 was adjusted for age, Model 2 included information in Model 1 plus childhood characteristics (parental mental illness and childhood physical illness), while Model 3 included the information in Model 2 plus SES in adulthood (educational attainment and annual household income). All analyses were stratified by gender. STATA MP 12 was used for the analysis.

## Results

### Characteristics of the sample population

Table 1 shows the mean ages of the men and women subjects were 50.1 (SE = 0.91) and 52.2 years (SE = 0.92) respectively, distributed normally. Regarding high SES in childhood, parental education was ≥13 years for 15.4% of the men and 11.7% of the women, although a significant portion of the participants did not know their parental educations (26.4% of the men and 28.3% of the women).

**Table 1:** Weighted distribution of characteristics by gender

			**Men (n = 734)**	**Women (n = 948)**	**p-value**
			**%**	**%**	
Demographics	Age	<30 years	13.9	15.1	0.26
		30-39 years	18.0	14.3	
		40-49 years	16.7	14.9	
		50-59 years	20.2	18.3	
		60-69 years	15.4	15.8	
		70-79 years	11.8	14.2	
		80+ years	4.0	7.4	
Socioeconomic status in childhood	Parental education	0-11 years	35.6	39.5	0.35
		12 years	22.6	20.5	
		13+ years	15.4	11.7	
		Unknown	26.4	28.3	
Childhood characteristics	Parental mental illness	Yes	2.3	2.7	0.62
	Physical illness	Yes	2.9	3.4	0.69
Socioeconomic status in adult	Education	0-11 years	25.5	31.1	<0.001
		12 years	31.2	33.2	
		13-15 years	15.4	24.3	
		16+ years	27.9	11.4	
	Annual household income	<3 million yen	26.8	36.6	<0.001
		3- < 10 million yen	54.9	52.0	
		10+ million yen	18.3	11.5	
Mental disorders	Major depression		4.7	8.6	<0.001
	Generalized anxiety disorder		2.8	3.0	0.83

In terms of childhood characteristics, less than 5% of respondents across both genders reported having parents with psychiatric illnesses or having their own physical illnesses in childhood. As for SES in adulthood, 27.9% of the men and 11.4% of the women graduated from college or achieved some other level of higher education. Further, 18.3% of the men and 11.5% of the women earned more than 10 million yen per year. Finally, 4.7% of the men and 8.7% of the women developed MD, while 2.8% of the men and 3.0% of the women developed GAD during their lifetimes.

### Association of SES with MD

Table 2 shows the ORs of childhood SES for MD among men. SES in childhood (i.e., parental education) was not associated with MD in Model 1 (adjusting for age), Model 2 (plus adjustment for childhood characteristics), or Model 3 (plus adjustment for SES in adulthood). Among the covariates, having a physical illness in childhood and a higher educational attainment (i.e., ≥16 years) were significantly independently associated with the onset of MD. That is, those who had physical illness in childhood were 2.89 (95% CI: 1.00-8.32) times more likely to develop MD than those who did not, and those who attained ≥16 years of education were 3.14 (95% CI: 1.08-9.14) times more likely to develop MD than those who attained 0–11 years of education.

**Table 2:** Odds ratio of socioeconomic status in childhood and covariates for major depression by discrete-time survival analysis, men

			**Model 1 (univariate, adjusted for age)**		**Model 2 (+childhood characteristics)**		**Model 3 (+SES in adult)**	
			**OR**	**95% CI**	**OR**	**95% CI**	**OR**	**95% CI**
SES in childhood	Parental education	0-11 years	ref		ref		ref	
		12 years	1.18	(0.51-2.76)	1.24	(0.54-2.86)	1.04	(0.48-2.25)
		13+ years	0.83	(0.32-2.18)	0.77	(0.29-2.06)	0.51	(0.19-1.34)
		Unknown	1.12	(0.50-2.53)	1.17	(0.51-2.64)	1.21	(0.52-2.78)
Childhood characteristics	Parental mental illness	Yes			2.23	(0.64-7.74)	2.00	(0.56-7.11)
		No			ref		ref	
	Physical illness	Yes			**2.90**	**(1.02-8.28)**	**2.89**	**(1.00-8.32)**
		No			ref		ref	
SES in adulthood	Education	0-11 years					ref	
		12 years					1.05	(0.35-3.18)
		13-15 years					1.59	(0.45-5.65)
		16+ years					**3.14**	**(1.08-9.14)**
	Annual household income	<3 million yen					ref	
		3- < 10 million yen					0.91	(0.39-2.09)
		10+ million yen					0.79	(0.31-2.02)

Age was adjusted for all analysis. Values in bold are significant at the p = 0.05 level.

In contrast, among women, high SES in childhood (i.e., parental education that went beyond high school), was positively associated with the onset of MD (Table 3), and this relationship was quite robust. Participants with high parental education were 1.85 (95% CI: 1.00-3.42) times more likely to develop MD than those whose parental education was lower than high school in Model 2, which was slightly attenuated in Models 3. Among other covariates, those who attained high school education were more likely to develop MD than those who attained education level lower than high school (OR: 2.39, 95% CI: 1.19–4.81).

**Table 3:** Odds ratio of socioeconomic status in childhood and covariates for major depression by discrete-time survival analysis, women

			**Model 1 (univariate, adjusted for age)**		**Model 2 (+childhood characteristics)**		**Model 3 (+SES in adult)**	
			**OR**	**95% CI**	**OR**	**95% CI**	**OR**	**95% CI**
SES in childhood	Parental education	0-11 years	ref		ref		ref	
		12 years	1.73	(0.97-3.09)	**1.80**	**(1.01-3.21)**	1.68	(0.97-2.92)
		13+ years	**1.84**	**(1.01-3.33)**	**1.85**	**(1.00-3.42)**	**1.81**	**(1.03-3.18)**
		Unknown	0.84	(0.52-1.37)	0.88	(0.54-1.44)	0.94	(0.56-1.55)
Childhood characteristics	Parental mental illness	Yes			**2.48**	**(1.16-5.32)**	2.17	(0.93-5.09)
		No			ref		ref	
	Physical illness	Yes			1.27	(0.51-3.19)	1.28	(0.50-3.32)
		No			ref		ref	
SES in adulthood	Education	0-11 years					ref	
		12 years					**2.39**	**(1.19-4.81)**
		13-15 years					1.95	(0.86-4.46)
		16+ years					2.45	(0.92-6.49)
	Annual household income	<3 million yen					ref	
		3- < 10 million yen					0.94	(0.60-1.47)
		10+ million yen					1.12	(0.59-2.14)

Age was adjusted for all analysis. Values in bold are significant at the p = 0.05 level.

### Association of SES with GAD

Table 4 shows the ORs of childhood SES for GAD among men. Higher parental education was significantly associated with the onset of GAD. Those whose parental education was high school or beyond high school were 5.63 (95% CI: 1.16–27.41) and 8.47 (95% CI: 1.87-38.37) times more likely to develop GAD, respectively, than those whose parental education was lower than high school in Model 1, which was slightly attenuated after adjusting for childhood characteristics and SES in adulthood (Model 3). In contrast to the results for MD, no association was found between the onset of GAD and childhood physical illness.

**Table 4:** Odds ratio of socioeconomic status in childhood and covariates for generalized anxiety disorder by discrete-time survival analysis, men

			**Model 1 (univariate, adjusted for age)**		**Model 2 (+childhood characteristics)**		**Model 3 (+SES in adult)**	
			**OR**	**95% CI**	**OR**	**95% CI**	**OR**	**95% CI**
SES in childhood	Parental education	0-11 years	ref		ref		ref	
		12 years	**5.63**	**(1.16-27.41)**	**5.63**	**(1.15-27.47)**	4.24 *	(0.96-18.74)
		13+ years	**8.47**	**(1.87-38.37)**	**8.55**	**(1.84-39.72)**	**6.84**	**(1.62-28.94)**
		Unknown	1.70	(0.40-7.20)	1.70	(0.40-7.20)	1.80	(0.42-7.72)
Childhood characteristics	Parental mental illness	Yes			1.15	(0.14-9.17)	1.11	(0.14-8.73)
		No			ref		ref	
	Physical illness	Yes			0.50	(0.07-3.69)	0.45	(0.06-3.37)
		No			ref		ref	
SES in adult	Education	0-11 years					ref	
		12 years					3.74	(0.62-22.49)
		13-15 years					2.85	(0.39-20.85)
		16+ years					3.54	(0.63-19.96)
	Annual household income	<3 million yen					ref	
		3- < 10 million yen					1.02	(0.29-3.62)
		10+ million yen					0.95	(0.20-4.51)

Age was adjusted for all analysis. *p = 0.057. Values in bold are significant at the p = 0.05 level.

On the other hand, among women, no association was found between childhood SES and the onset of GAD (Table 5). Moreover, no other covariates had any significant association with the onset of GAD, including SES in adulthood.

**Table 5:** Odds ratio of socioeconomic status in childhood and covariates for generalized anxiety disorder by discrete-time survival analysis, women

			**Model 1 (univariate, adjusted for age)**		**Model 2 (+childhood characteristics)**		**Model 3 (+SES in adult)**	
			**OR**	**95% CI**	**OR**	**95% CI**	**OR**	**95% CI**
SES in childhood	Parental education	0-11 years	ref		ref		ref	
		12 years	0.40	(0.15-1.06)	0.42	(0.16-1.13)	0.40	(0.14-1.13)
		13+ years	1.39	(0.59-3.26)	1.34	(0.52-3.44)	1.27	(0.50-3.27)
		Unknown	0.85	(0.32-2.30)	0.92	(0.33-2.57)	0.85	(0.29-2.50)
Childhood characteristics	Parental mental illness	Yes			3.25	(0.79-13.34)	2.47	(0.42-14.38)
		No			ref		ref	
	Physical illness	Yes			2.64	(0.51-13.76)	3.11	(0.59-16.50)
		No			ref		ref	
SES in adult	Education	0-11 years					ref	
		12 years					0.64	(0.21-2.00)
		13-15 years					0.41	(0.08-2.17)
		16+ years					0.94	(0.17-5.20)
	Annual household income	<3 million yen					ref	
		3- < 10 million yen					0.48	(0.22-1.05)
		10+ million yen					0.61	(0.21-1.81)

Age was adjusted for all analysis.

We also estimated our model excluding unknown parental education cases in order to complete a sensitivity analysis. No substantial change in our results was found.

## Discussion

Unlike what has been found in previous studies in Western societies [8-13], we found that, among women, higher SES in childhood is positively associated with the onset of MD, but not GAD, even after adjusting for age, childhood characteristics, and SES in adulthood. In contrast, higher childhood SES among men is associated with GAD, but not with MD, after fully adjusting for other covariates. High SES in adulthood, represented as educational attainment, is also positively associated with MD for both genders.

Our results indicate that high SES in childhood has a direct effect on the onset of mental disorders in Japan. Previous studies on SES and mental disorders in Japan have reported inconsistent results; that is, higher educational attainment may [35] or may not be [16,17] associated with mental disorders. In our study, high childhood SES was positively associated with the onset of mental disorders (more precisely, MD and GAD); however, the exact mechanism of this positive association is unknown. Asian parents tend to have stronger expectations for their children [36,37] in terms of educational achievements than do Western parents [38]. Similarly, Japanese parents, particularly those in higher SES families, have high expectations for their children [39,40]. Therefore, it is likely that those who come from high parental SES situations may feel more pressure to achieve; thus, they may feel distressed when they fail to do so into adulthood. Moreover, those who come from a high-SES family may have been overprotected during childhood, a phenomenon that has been shown to induce lower stress tolerance [41,42]. Thus, when they encounter stressful academic, professional, or social situations, they are more likely to develop mental disorders.

The impact of high SES in childhood has specific associations by gender and disorder. High childhood SES is associated with MD only among women, and it is associated with GAD only among men. This is probably due to gender differences in stress response [43]. Women tend to internalize stress and feel disappointment or decreased self-esteem when they face stressful situations [44-46]. Thus, women who experienced high SES in childhood are more likely to develop MD. Meanwhile, men with higher SES in childhood might feel more pressure and a heightened sense of personal responsibility when they enter middle age, resulting in the development of GAD. Previous studies have shown that childhood SES is positively associated with average levels of educational and occupational expectations throughout adulthood [47,48]. Furthermore, qualitative study is needed to confirm how women or men with high childhood SES deal with that stress.

Our results showed that respondents’ educational attainment had independent associations with MD, regardless of gender. The directionality is unknown; that is, whether higher educational attainment is the cause of MD, or if MD induces higher educational attainment (although this is highly unlikely). Nonetheless, it is noteworthy to mention that childhood SES is independently associated with MD, regardless of SES in adulthood (i.e., educational attainment).

Several limitations of the current study suggest avenues for future research. First, this study used self-reports of SES in childhood and parental mental illness, rather than a direct assessment of the respondents’ parents. However, previous studies that also used self-reported childhood SES [13] have found similar results [11]. Second, it is possible that we overestimated the association between childhood SES and mental disorders because of common method bias—that is, participants who have stressful memories related to parental SES might have been more likely to report symptoms of mental disorders. Third, although this study was population-based, and weighted analysis was used to adjust for the differences in demographic variables between the respondents and non-respondents, the comparatively small study sample size may not be representative of the whole Japanese population. Further investigation using a larger, nationally representative sample is warranted.

## Conclusion

In Japan, childhood SES is likely to be positively associated with the lifetime onset of mental disorders, regardless of family history of mental disorders, childhood physical illness, or SES in adulthood. Further study is needed to replicate these findings and to elucidate other factors, such as parental pressures or social expectations.

## Competing interests

The authors declare that they have no conflict of interest.

## Authors’ contributions

MO was involved in the literature review and the drafting of the manuscript. TF conceived the study hypothesis, performed the statistical analyses, and wrote the first draft, and. RM helped to performed the statistical analyses and draft the manuscript. NK critically evaluated and revised the manuscript to ensure the inclusion of important intellectual content. All the authors read and approved the final manuscript.

## Pre-publication history

The pre-publication history for this paper can be accessed here:

http://www.biomedcentral.com/1471-2458/14/359/prepub
